# Effects of Hyeonggaeyeongyo-Tang in Ovalbumin-Induced Allergic Rhinitis Model

**DOI:** 10.1155/2014/418705

**Published:** 2014-06-26

**Authors:** Se Hyang Hong, Soon Re Kim, Han-Seok Choi, Jin Mo Ku, Hye Sook Seo, Yong Cheol Shin, Seong-Gyu Ko

**Affiliations:** Laboratory of Clinical Biology and Pharmacogenomics, Department of Preventive Medicine, College of Oriental Medicine, Kyung Hee University, Seoul 130-701, Republic of Korea

## Abstract

Allergic rhinitis (AR) is an allergic inflammation of the nasal airways. The prevalence of AR is increasing worldwide. We investigated whether Hyeonggaeyeongyo-tang (HYT) is effective to suppress the progression of AR induced by ovalbumin (OVA). Male BALB/c mice were used for this study. Allergic rhinitis was induced by OVA. Treatment with HYT was assessed to study the effect of HYT on allergic rhinitis in mice. Histological analysis, immunohistochemistry, multiplex cytokine assay, blood analysis, and cell viability assay were performed to verify inhibitory effect of HYT on allergic rhinitis. HYT did not show any toxicity maintaining body weight. Food intake was steady without variation in mice. HYT reduced infiltration of inflammatory cells and mast cells into nasal cavity. HYT reduced the levels of cytokines and leukocytes in the blood. HYT decreased the splenocyte cell viability. Antihistamines and steroids are the most common medications used to treat allergic rhinitis. However, long-term use of drug generates resistance or side effects requiring the development of new drug. Our present study clearly demonstrates that HYT suppresses the progression of allergic rhinitis induced by OVA. This suggests that HYT might be a useful drug for the treatment of allergic rhinitis.

## 1. Introduction

Allergic rhinitis (AR) is an allergic inflammation of the nasal airways and is characterized by sneezing, nasal congestion, nasal itching, and rhinorrhea, in any combination. The prevalence of AR is increasing worldwide. AR affects approximately 60 million people in the United States and the prevalence is about 10–30% in adults and nearly 40% in children [1–3]. A nationwide questionnaire survey conducted with 42,886 Koreans using the International Study of Asthma and Allergies in Childhood (ISSAC) questionnaire demonstrated that 12-month prevalence of 6–12 and 12–15 years of age with AR were 28.8% and 29.1%, respectively [4].

Patients with AR present an inflammatory IgE-mediated response characterized by allergen type 2 helper T cells (Th2) immunological pattern with mast cells and eosinophil activation and release of inflammatory mediators in response to exposure to allergens [5–7]. Allergens are any substance, most often eaten or inhaled, that can be found in a variety of sources, such as dust, pollen, and pet dander. Such allergens (antigens) induce eosinophil recruitment into the tissues and increase serum IgE level [8]. Mast cells contain many granules rich in histamine and heparin. In allergic reactions, mast cells become active when an allergen binds to IgE [9, 10]. In addition, not only mast cells but also B cells play an important role in the pathogenesis of AR [11–16]. Allergen type 2 helper T cells (Th2) play a major role in the regulation of IgE antibody producing B cells, mast cells, and eosinophils. Moreover, Th2 secrete IL-4, IL-5, IL-6, and IL-13 and regulate B cell and eosinophil mediated responses, whereas Th1 produce interferon. Th1/Th2 cells are associated with a series of immune and inflammatory diseases, such as bacterial and viral infectious diseases [17, 18]. T cell-mediated immunity is an important process of elaborating antigen (Ag) specific T lymphocytes to remove viral, bacterial, or parasitic infections or malignant cells. Cytotoxic CD8+ T cells are T lymphocytes that kill infected, damaged, or malignant cells bearing the Ag, while CD4+ T helper cells generate cytokines that can be directly poisonous to the target cells or can stimulate other T cell effector functions and B cell antibody production and mobilize powerful inflammatory mechanisms [19].

The traditional herbal medicine Hyeonggaeyeongyo-tang (HYT) is known to treat otitis media, sinusitis, tonsillitis, and a variety of otolaryngology symptoms [20]. In Japan and China, HYT is known as Keigai-rengyo-to or Jing Jie Lian Qiao Tang, respectively. However, its effect on AR has not yet been elucidated. Antihistamines and steroids are the most common medications used to treat AR [21]. However, long-term use of drug generates resistance or side effects requiring the development of new drug [22].

Therefore, in this study, we investigated the effects of HYT on allergic responses in ovalbumin- (OVA-) induced AR mice model. For that purpose, we performed histopathological analysis and measured mast cell numbers. We also measured the levels of cytokines in the serum and the level of leukocytes in the blood. Moreover, we evaluated splenocyte viability.

## 2. Materials and Methods

### 2.1. Preparation of Hyeonggaeyeongyo-Tang (HYT)

HYT used in this study contains thirteen different herbal medicines:* Schizonepeta Spica* 0.47 g,* Forsythiae Fructus* 0.47 g,* Saposhnikoviae Radix* 0.93 g,* Angelicae Gigantis Radix* 0.93 g,* Cnidii Rhizoma* 0.93 g,* Paeoniae Radix Alba* 0.93 g,* Bupleuri Radix* 0.47 g,* Aurantii Fructus* 0.93 g,* Scutellariae Radix* 0.93 g,* Gardeniae Fructus* 0.93 g,* Angelicae Dahuricae Radix* 0.93 g,* Platycodi Radix* 0.93 g, and* Glycyrrhizae Radix* 0.67 g. They were prepared by Hanpoong Pharm (Jeonju, Republic of Korea) following good manufacturing practice (GMP) procedure. Dried HYT powder was stored in aliquots at −80°C until further analysis (Table 1).

### 2.2. Animals

Male BALB/c mice (aged 6 weeks) were purchased from Orient (Seoul, Republic of Korea). The average body weight of mice was 26 g. The animals were housed in air-conditioned room with a 12 h light/dark cycle. All mice were allowed free access to food and water. All procedures performed on the mice were approved by the animal care center of Kyung Hee University (Approval number KHUASP (SE)-12-048). The mice were divided into five groups (*n* = 8): group 1, normal saline; group 2, OVA + saline; group 3, OVA + HYT (101 mg/kg); group 4, OVA + HYT (202 mg/kg); group 5, OVA + HYT (404 mg/kg).

### 2.3. Sensitization and Treatment

**Figure 1:**
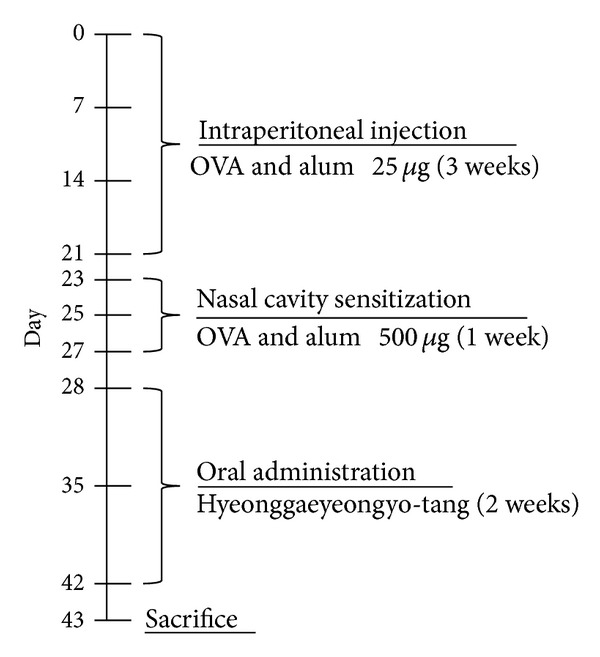
Allergic rhinitis model.

The experimental procedures for allergic sensitization and challenge are summarized in Figure 1. For establishment of OVA-induced AR model, male 6-week-old BALB/c mice were intraperitoneally sensitized with 25 ug of OVA twice a week for three weeks. After three weeks, OVA group of mice were sensitized by intranasal instillation of 500 ug OVA in 30 uL PBS twice a week for one week. Finally, mice were fed with HYT together with OVA sensitization for 2 weeks. At the end of experiment, mice were killed by CO2 inhalation, and samples were collected.

### 2.4. Histological Analysis

Nasal mucosa samples were immediately fixed with 10% formaldehyde and embedded in paraffin. The sections of the nasal mucosa samples were 4-*μ*m thick. Each section was stained with hematoxylin and eosin (H & E) for inflammatory cells or with toluidine blue (T.B) for mast cells counts and examined under light microscopy (Olympus). Mast cells and inflammatory cells were counted in 10 parts of high power fields (HPF) at 40x, 400x, and 1000x magnification.

### 2.5. Immunohistochemistry

Expression of CD4+ lymphocytes was detected by immunohistochemical analysis using the anti-CD4+ antibody. The nasal tissues were deparaffinized and rehydrated. After a microwave treatment, the sections were treated with 3% hydrogen peroxide in PBS for 15 min to inhibit endogenous peroxidase activity of blood cells. Following hydrogen peroxide treatment, the nasal sections were incubated with 5% bovine serum albumin (BSA) in PBS, contained a blocking reagent for 1 hour at room temperature. Nasal sections were incubated with mouse monoclonal CD4+ antibody overnight at 4°C and subsequently incubated with secondary biotinylated anti-rabbit IgG for 1 h at room temperature. Sections were treated with avidin-biotin HRP complex (Vectastain ABC kit, Vector Labs, CA, USA) for 30 min at 4°C and stained with diaminobenzidine tetrachloride (DAB) as the substrate. The slides were mounted with an aqueous mounting solution (DAKO, Glostrup, Denmark) and cover-slipped. All the sections were analyzed using an Olympus microscope and images were captured using a digital video camera.

### 2.6. Multiplex Cytokine Assay

Blood serum was analyzed by the Bio-Plex multiplex cytokine assay (Bio-Rad Laboratories, Hercules, CA, USA) according to the manufacturer's instructions. The assay was read using a Luminex 100 (Austin, TX) and analyzed using a Bio-Plex Manager software. The mean concentration of cytokines (IL-4, IL-13, and LIF) in supernatants from OVA-stimulated cells over the unstimulated cells (background) was then calculated.

### 2.7. Blood Analysis

Whole blood samples were collected by cardiac puncture. The blood was placed in Vacutainer TM tubes containing EDTA (BD science, USA). Anticoagulated blood was processed to determine hematological parameters (WBC, lymphocytes, monocytes, eosinophils, basophils, and neutrophils) in a HEMAVET 950 hematology analyzer (Drew Scientific, Inc., Oxford) in accordance with manufacturer' recommendation.

### 2.8. Splenocytes

Suspension of spleen from normal mice under aseptic condition was prepared by homogenization in RPMI-1640 medium (containing 10% FBS, 1% Ab, and 0.05 mM Mercaptoethanol). The suspension was centrifuged and pelleted. The contaminating red blood cells were removed by using red blood cell lysis buffer (Sigma, USA). Cells were centrifuged and suspended in complete RPMI-1640. Cells were maintained at 37°C in a humidified incubator with 5%.

### 2.9. Cell Viability Assay

Mice splenocytes (1 × 10^6^ cells/well) were plated in 96-well culture plates and incubated for 24 h. Cells were treated with Con A (2 ug/mL) or HYT (10, 50, 100, 200, 500, 1000, and 2000 ug/mL). After 24 h incubation, 10 uL of WST solution was added to each well of the plate, and the plates were incubated in the dark at 37°C for another 2 h. Optical density was measured at 450 nm using an ELISA plate reader (VersaMax, Molecular Devices, CA, USA).

### 2.10. Statistical Analysis

All experiment results were expressed as the means ± standard deviations (SD) or means ± SEM (*n* = 8) of at least three separate tests. Student's *t*-test was used for single variable comparisons, and a *P* value < 0.05 was considered statistically significant.

## 3. Results

### 3.1. Body Weight and Food Intake

**Figure 2:**
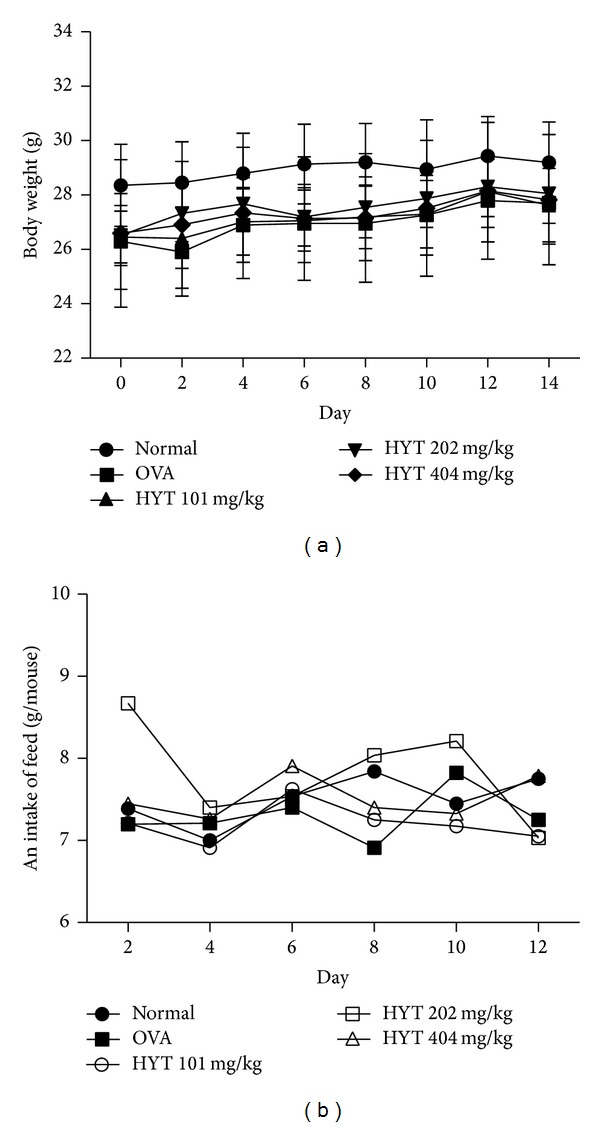
Changes in body weight (a) and food intake (b) during treatments with HYT in OVA-induced AR mice model. Values are expressed as mean ± SEM (*n* = 8).

Body weight and food intake were monitored throughout the study. We found that normal group shows higher weight as compared to other groups (Figure 2(a)). We also found that HYT did not show any toxicity maintaining body weight (Figure 2(a)). In addition, food intake was steady without variation (Figure 2(b)).

### 3.2. HYT Reduced Infiltration of Inflammatory Cells into Nasal Cavity

**Figure 3:**
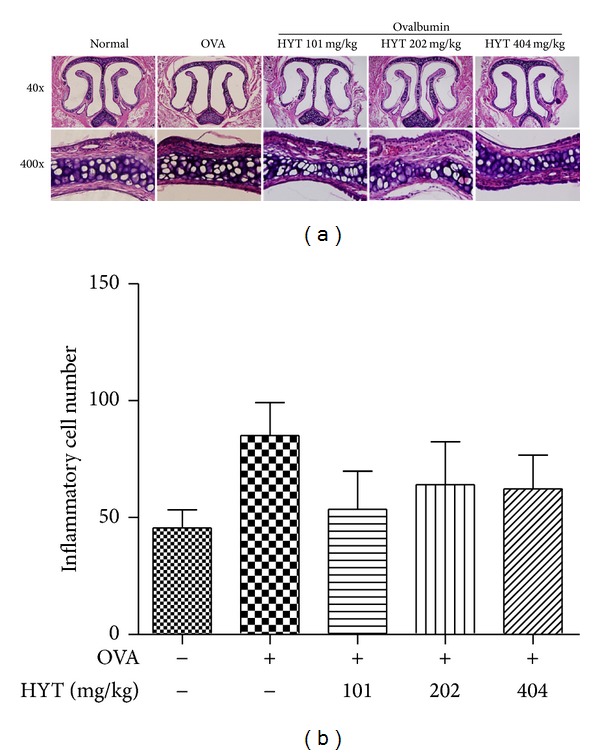
HYT reduced infiltration of inflammatory cells into nasal cavity. The nasal mucosa sections were stained with hematoxylin and eosin. Sections were evaluated using microscope at an original magnification of 40x and 400x. Data are presented as mean ± SEM.

To determine whether HYT reduces infiltration of inflammatory cells into nasal cavity, we performed H & E staining on the nasal mucosa samples. The respective numbers of inflammatory cells in the nasal mucosa in AR mice model were shown to be higher than those in the normal mice. HYT decreased such infiltration of inflammatory cells into nasal cavity (Figure 3(a)). Inflammatory cell numbers under each condition were shown in Figure 3(b).

### 3.3. HYT Reduced Infiltration of Mast Cells into Nasal Mucosa

**Figure 4:**
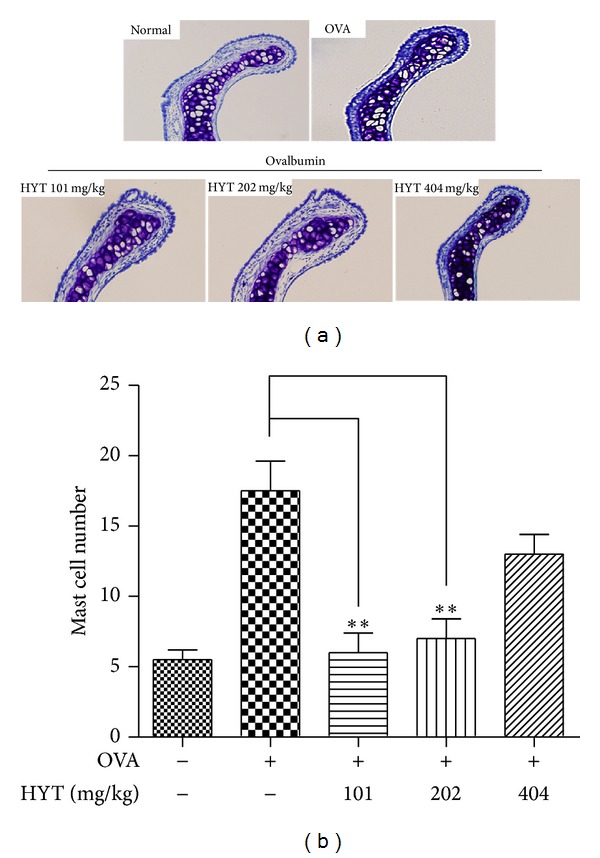
HYT reduced infiltration of mast cells into nasal mucosa. The nasal mucosa sections were stained with toluidine blue. Sections were evaluated using microscope at an original magnification of 200x. Data are presented as mean ± SEM. *P < 0.05, **P < 0.01, and ***P < 0.001.

Next, we performed toluidine blue staining for mast cell observation. Mast cells play a major role in allergic inflammation [23]. The respective numbers of mast cells in the nasal mucosa in AR mice were shown to be higher than those in the normal mice. HYT decreased such infiltration of mast cells into nasal cavity (Figure 4(a)). Lowest HYT concentration showed the highest effect to reduce infiltration of mast cells into nasal mucosa. Mast cell numbers under each condition were shown in Figure 4(b).

### 3.4. HYT Reduced Expression of CD4+ Cells into Nasal Cavity

**Figure 5:**
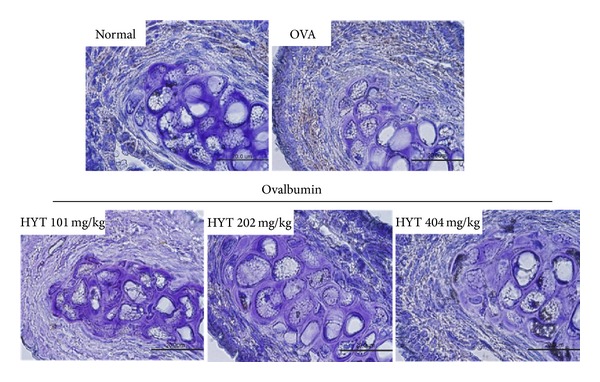
Distribution of CD4+ cells in nasal cavity samples. The nasal cavity sections were immunostained with CD4+ antibody. CD4+ cells show a brown color. Sections were evaluated using microscope at an original magnification of 1000x.

CD4+ T helper cells generate cytokines that can stimulate other T cell effector functions and B cell antibody production [19]. We also performed immunocytochemistry to measure intracellular level of CD4+ (total T cells). We found that OVA increased numbers of CD4+ (total T cells), while HYT decreased them in nasal cavity (Figure 5).

### 3.5. HYT Reduced the Levels of Cytokines in the Serum

**Figure 6:**
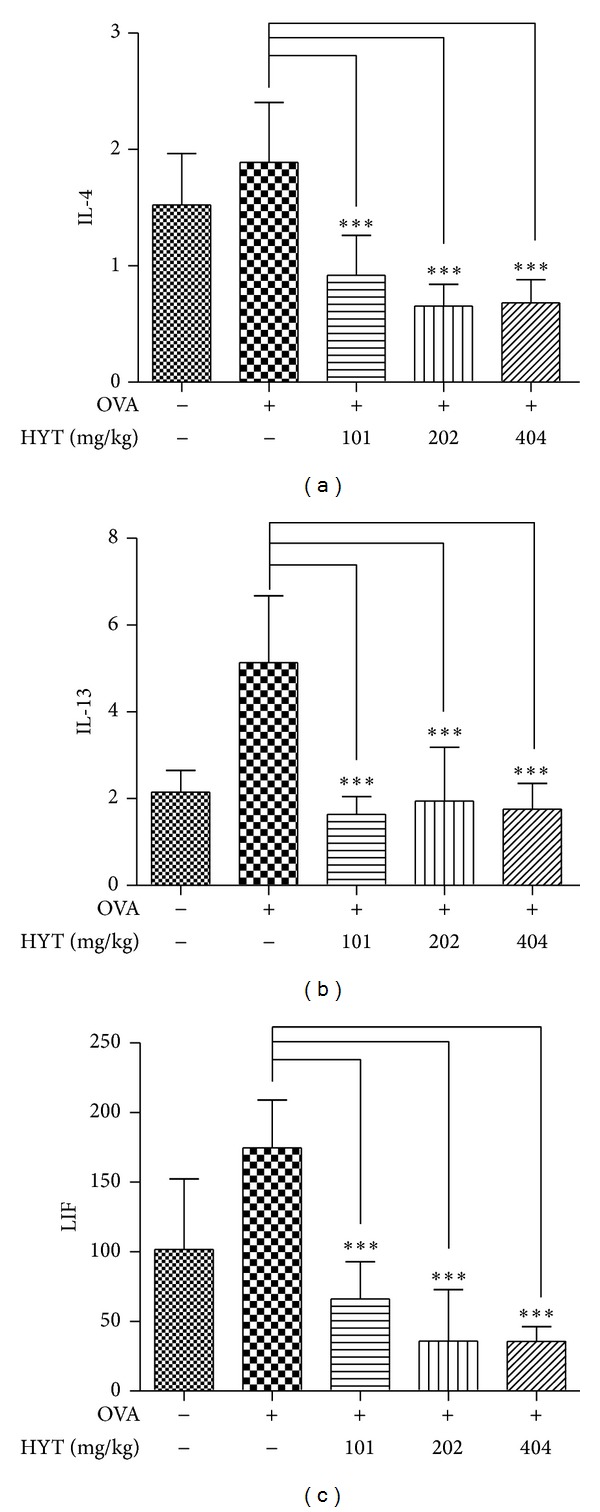
HYT reduced the levels of cytokines in the serum. The cytokines levels were measured by multiplex cytokine assay. Data are presented as mean ± SEM. *P < 0.05, **P < 0.01, and ***P < 0.001.

Allergic reactions cause the secretion of various cytokines [23]. Therefore, we measured the levels of cytokines in the blood samples by multiplex cytokine assay. We found that OVA increases the levels of IL-4, IL-13, and LIF, while HYT inhibits such increase (Figures 6(a), 6(b), and 6(c)).

### 3.6. HYT Reduced the Levels of Leukocytes in the Blood

**Figure 7:**
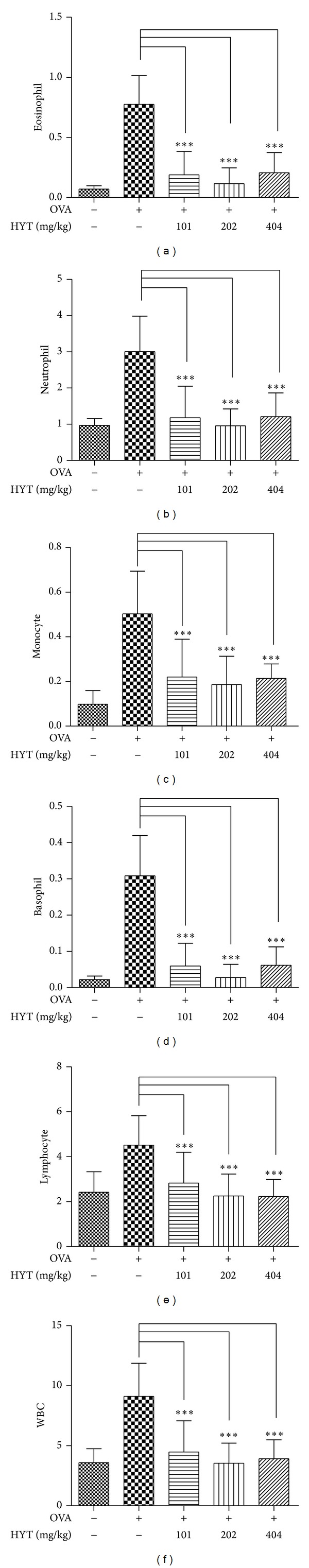
HYT reduced the levels of leukocytes in the blood. Blood samples were analyzed using HEMAVET 950 hematology analyzer. Data are presented as mean ± SEM. *P < 0.05, **P < 0.01, and ***P < 0.001.

It is known that allergic diseases activate eosinophils, neutrophils, monocytes, basophils, and lymphocytes [24, 25]. To investigate whether HYT suppresses inflammatory phenomenon, we measured leukocytes levels in cardiovascular blood samples using HEMAVET 950 hematology analyzer. We found that OVA increases the levels of eosinophils, neutrophils, monocytes, basophils, lymphocytes, and WBC, while HYT inhibits such increase (Figures 7(a), 7(b), 7(c), 7(d), 7(e), and 7(f)).

### 3.7. Effect of HYT on Splenocyte Viability

**Figure 8:**
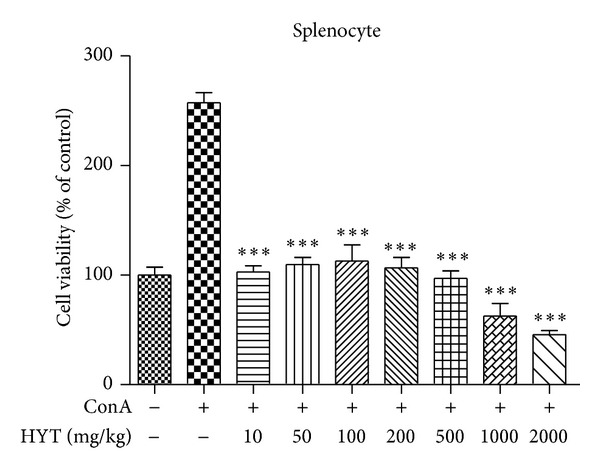
Effect of HYT on splenocyte viability. Splenocytes were treated with various concentrations of HYT (10, 50, 100, 200, 500, 1000, and 2000 ug/mL). Cell viability was measured by WST assay. The columns and the error bars represent mean ± standard deviation (SD). *P < 0.05, **P < 0.01, and ***P < 0.001.

Finally, we measured the effect of HYT on splenocyte viability. For that purpose, we treated splenocyte with various concentrations of HYT (10, 50, 100, 200, 500, 1000, and 2000 ug/mL) and measured cell viability using WST assay. We found that con A increases cell viability, while HYT decreases it (Figure 8).

## 4. Discussion

In this study, we investigated the effect of HYT on allergic responses in OVA-induced AR mice. Increase of infiltration of inflammatory and mast cells into nasal cavity is known in OVA-induced AR mice. Interestingly, HYT strongly suppressed such increase. Immunohistochemical study showed thatHYT reduced increased number of CD4+ T cells induced by OVA. Moreover, induction of various cytokines due to AR was also suppressed by HYT in mice.

The activation of eosinophils releases a variety of chemicals to cause inflammation and tissue injuries [26, 27]. A variety of cytokines are released by inflammatory stimulation [28–30]. We found that OVA increases the levels of IL-4, IL-13, and LIF, while HYT inhibits such increase. We also found that OVA increases the levels of eosinophils, neutrophils, monocytes, basophils, lymphocytes, and WBC, while HYT inhibits such increase. HYT also decreased cell viability of splenocyte. These data clearly demonstrate that HYT has an efficacy to treat AR.

AR, also known as hay fever, affects approximately 20 percent of people of all ages. The risk of developing AR is much higher in people with asthma or eczema and in people who have a family history of asthma or rhinitis [31]. AR can begin at any age, although most people first develop symptoms in childhood or young adulthood [4]. The symptoms are often at their worst in children and in people in their 30s and 40s. The severity of symptoms tends to vary throughout life; many people experience periods when they have no symptoms at all [32].

The treatment of AR includes reducing exposure to allergens and other triggers, in combination with medication therapy. Nasal glucocorticoids (steroids) delivered by a nasal spray are the first-line treatment for the symptoms of AR. However, we require more effective therapy to treat AR with few side effects. HYT is known to treat otitis media, sinusitis, tonsillitis, and a variety of otolaryngology symptoms [20]. In this study, we verified that HYT could be used to treat AR. Our present study clearly demonstrates that HYT suppresses the progression of AR induced by OVA. This suggests that HYT might be a useful drug for the treatment of AR.

## Figures and Tables

**Table 1:** The composition of herbal medicines in HYT.

Composition	Volume
*Schizonepeta Spica *	0.47 g
*Forsythiae Fructus *	0.47 g
*Saposhnikoviae Radix *	0.93 g
*Angelicae Gigantis Radix *	0.93 g
*Cnidii Rhizoma *	0.93 g
*Paeoniae Radix Alba *	0.93 g
*Bupleuri Radix *	0.47 g
*Aurantii Fructus *	0.93 g
*Scutellariae Radix *	0.93 g
*Gardeniae Fructus *	0.93 g
*Angelicae Dahuricae Radix *	0.93 g
*Platycodi Radix *	0.93 g
*Glycyrrhizae Radix *	0.67 g
